# Oxalate Activates Autophagy to Induce Ferroptosis of Renal Tubular Epithelial Cells and Participates in the Formation of Kidney Stones

**DOI:** 10.1155/2021/6630343

**Published:** 2021-10-06

**Authors:** Qianlin Song, Wenbiao Liao, Xin Chen, Ziqi He, De Li, Bin Li, Junwei Liu, Lang Liu, Yunhe Xiong, Chao Song, Sixing Yang

**Affiliations:** ^1^Department of Urology, Renmin Hospital of Wuhan University, Wuhan, China; ^2^Reproductive Medical Center, Renmin Hospital of Wuhan University and Hubei Clinic Research Center for Assisted Reproductive Technology and Embryonic Development, Wuhan, China

## Abstract

Renal tubular epithelial cell damage is the basis for the formation of kidney stones. Oxalate can induce human proximal tubular (HK-2) cells to undergo autophagy and ferroptosis. The present study was aimed at investigating whether the ferroptosis of HK-2 cells induced by oxalate is caused by the excessive activation of autophagy. We treated HK-2 cells with 2 mmol/L of oxalate to establish a kidney stone model. First, we tested the degree of oxidative damage and the level of autophagy and ferroptosis in the control group and the oxalate intervention group. We then knocked down and overexpressed the *BECN1* gene and knocked down the *NCOA4* gene in HK-2 cells, followed by redetection of the above indicators. We confirmed that oxalate could induce autophagy and ferroptosis in HK-2 cells. Moreover, after oxalate treatment, overexpression of the *BENC1* gene increased cell oxidative damage and ferroptosis. In addition, knockdown of *NCOA4* reversed the effect of oxalate-induced ferroptosis in HK-2 cells. Our results show that the effects of oxalate on the ferroptosis of HK-2 cells are caused by the activation of autophagy, and knockdown of the *NCOA4* could ameliorate this effect.

## 1. Introduction

Kidney stones are mineral polymers that are free from the renal pelvis and calyces or are deposited in the renal papilla. Kidney stones are one of the most common diseases in urology, representing a burden on global public health [1, 2]. Epidemiological surveys show that the global incidence of kidney stones is 1.7–14.8%, and the 5-year recurrence rate of kidney stones can be as high as 50% [3]. The high rate of recurrence of calculi has resulted in kidney stones being considered a systemic chronic disease [4]. Unfortunately, although the surgical treatment of kidney stones and surgical instruments have developed rapidly in recent years, these advanced surgical treatments are not effective in reducing the high incidence and recurrence rate of kidney stones [4, 5]. Therefore, it is necessary to explore the formation mechanism of kidney stones and determine the etiology and treatment of stones to effectively prevent their recurrence.

Damage to renal tubular epithelial cells and the supersaturation of crystals in urine are the two major foundations for the formation of kidney stones [6, 7]. High concentrations of oxalate can cause damage to proximal and distal kidney tubular cells, and oxalate is also the most important factor in crystal aggregation [8–10]. Autophagy is a highly conserved process used to degrade and recycle biological macromolecules or damaged organelles and is involved in a variety of human diseases [11]. BECN1, also known as ATG6 or beclin1, is a key molecule that regulates autophagy activity. BECN1 is involved in the assembly of the BECN1-PIK3C3-PIK3R4 complex, which can trigger the autophagy protein cascade reaction and mediate the transport process of vesicles [12]. BECN1 can promote ferroptosis of various cancer cells by blocking the activity of system X_c_^−^ [13]. Studies have shown that hyperoxaluria and calcium oxalate crystals will continue to stimulate renal tubular epithelial cells, causing excessive activation of autophagy, thus leading to cell damage and death [14]. Ferroptosis is an iron-dependent cell death method characterized by loss of glutathione peroxidase 4 (GPX4) activity and lipid peroxide deposition [15, 16]. Our previous studies confirmed that high concentrations of oxalate can mediate the ferroptosis of renal tubular epithelial cells, thereby participating in the formation of kidney stones [17]. There is a close relationship between autophagy and ferroptosis. Some researchers even believe that ferroptosis is a type of autophagy-dependent cell death [18]. The mechanism that induces ferroptosis through the activation of autophagy exists in many diseases, such as chronic arsenic poisoning [19], heart failure [20], and chronic obstructive pulmonary disease [21]. Nuclear receptor coactivator 4 (NCOA4) is a selective cargo receptor that can mediate ferritin phagocytosis to control the release and storage of intracellular iron, thereby maintaining the body's iron homeostasis [22]. According to previous studies, both autophagy and ferroptosis play an important role in the formation of kidney stones; however, it has not been determined whether there is a relationship between them and whether NCOA4 plays an important role in this relationship. Therefore, the present study was aimed at exploring the mechanism of autophagy-ferroptosis in oxalate-mediated renal tubular epithelial cell damage, to provide new ideas for the prevention and treatment of kidney stones.

## 2. Materials and Methods

### 2.1. Cell Culture and Grouping

We used the human proximal tubular cell line HK-2 (Cell Bank of the Chinese Academy of Sciences, Shanghai, China) for the experiments. The cells were cultured in Dulbecco's modified Eagle's medium (DMEM)/F-12 complete medium (DMEM/F-12 basic medium+10% fetal bovine serum (FBS)+1% penicillin) at 37°C and 5% CO_2_ in a cell culture incubator (Binder, Tuttlingen, Germany). First, to select the best concentration of oxalate (Sigma, Neustadt, Germany), we detected the changes of autophagy marker proteins and cell viability in HK-2 cells after treatment with different concentrations of oxalate (0.125, 0.25, 0.5, 1, and 2 mmol/L) intervention. Then, to confirm whether HK-2 cells underwent autophagy and ferroptosis under oxalate treatment, we tested the degree of oxidative damage and the level of autophagy and ferroptosis in the NC group (basic medium) and the Ox group (basic medium+2 mmol/L of oxalate). Then, to confirm whether the ferroptosis of HK-2 cells mediated by oxalate was caused by the activation of autophagy, we detected LDH (lactate dehydrogenase), MDA (malondialdehyde), GSH (glutathione), the Fe^2+^ content, ROS (reactive oxygen species), and ferroptosis-related proteins in the control NC (empty vector) group, the control Ox (empty vector+2 mmol/L of oxalate) group, the NC+BECN1-shRNA (*BECN1* knockdown plasmid) group, the Ox+BECN1-shRNA (*BECN1* knockdown plasmid+2 mmol/L of oxalate) group, the NC+BECN1 (*BECN1* overexpression plasmid) group, and the Ox+BECN1 (*BECN1* overexpression plasmid+2 mmol/L of oxalate) group. Next, to verify the role of NCOA4 in oxalate-mediated ferroptosis and autophagy in HK-2 cells, we detected the expression of NCOA4 in the stone model, and we detected MDA, GSH, the Fe^2+^ content, ROS, and ferroptosis-related proteins in the control NC group, the control Ox group, the NC+BECN1 group, the Ox+BECN1 group, the NC+BECN1+NCOA4-shRNA (*BECN1* overexpression plasmid+*NCOA4* knockdown plasmid) group, and the Ox+BECN1+NCOA4-shRNA (*BECN1* overexpression plasmid+*NCOA4* knockdown plasmid+2 mmol/L) group. These groups were cultured in basic medium, with an intervention time of 24 hours.

### 2.2. Animal Experiments

All animal experiment protocols were approved by the Animal Care Committee of Wuhan University (Wuhan, China), and the Laboratory Animal Welfare and Ethics Committee of Renmin Hospital of Wuhan University approved the study (IACUC Issue No. 20181212). We collected the animal specimen wax blocks preserved from previous studies [17]. The steps to establish the stone model are as follows: five-week-old male SD (Sprague-Dawley) rats (130–180 g) were used as experimental subjects. The control group had a normal diet, and the stone model group drank water containing 0.75% ethylene glycol. After feeding for one month, rats were sacrificed, and their kidneys were removed and subjected to silver nitrate staining, immunohistochemistry, and western blotting on the specimens of the normal control group and the kidney stone model group to explore the expression of NCOA4 in kidney stone model rats.

### 2.3. Plasmid Construction and Transfection

The pSuper-puro-BECN1-shRNA plasmid, the pEnCMV-BECN1 plasmid, and the empty vector were purchased from MiaoLing Plasmid Sharing Platform (Wuhan, China). The pSuper-puro-NCOA4-shRNA plasmid was purchased from Vigene Biosciences (Shandong, China). When the cell density reached 70–80%, the medium was replaced with basic medium. According to the manufacturer's instructions, the plasmid was mixed with Lipofectamine™ 2000 Transfection Reagent (11668019, Thermo Fisher, Waltham, MA, USA), added to cells, and incubated in a 37°C incubator for 5 hours, after which the cell medium was replaced with complete medium. Follow-up intervention and experimental testing were carried out after 24 hours.

### 2.4. Cell Viability Test

We use a Cell Counting Kit-8 (CCK8, Dojindo, Kumamoto, Japan) to test cell viability. The kit is based on WST-8, which can be reduced by a dehydrogenase in the mitochondria of living cells to produce orange-yellow compounds; the more the cells proliferate, the more obvious the orange-yellow. After cell intervention, the cell culture medium was removed, the cells were washed with phosphate-buffered saline (PBS) once, and the prepared CCK8 reagent (10 *μ*L CCK8+90 *μ*L DMEM/F-12 basic medium) was added. After incubating at 37°C for 2 hours, the absorbance of each well was detected at a wavelength of 450 nm using a microplate reader (PerkinElmer, Waltham, MA, USA).

### 2.5. LDH Detection

An LDH kit (Beyotime, Jiangsu, China) was used to detect LDH. After cell intervention, the corresponding reagents were added according to the manufacturer's instructions. The detailed steps are described in our previous study [3]. A microplate reader was used to detect the absorbance of each well at a wavelength of 490 nm.

### 2.6. MDA Detection

An MDA detection kit (Nanjing Jiancheng, Nanjing, China) was used to detect cell MDA levels. After cell intervention, the corresponding reagents were added according to the manufacturer's instructions, and the reaction was heated in a water bath at 95°C for 40 minutes. It was then cooled with running water and centrifuged at 4000 × *g* for 10 minutes, and then, the absorbance of each well was detected at a wavelength of 530 nm using a microplate reader.

### 2.7. GSH Detection

A GSH detection kit (Nanjing Jiancheng) was used for cell GSH detection. After cell intervention, the medium was removed and the cells were washed once with PBS. PBS was added and the cells were scraped into a centrifuge tube. The cells were broken by sonication. The working solution was added, mixed, and allowed to stand for 5 minutes; after which, the absorbance of each well was measured at 405 nm wavelength using a microplate reader.

### 2.8. ROS Detection

We used a ROS detection kit (Beyotime, China) to detect the ROS in the samples. After intervention, the cell sample was washed once with PBS. The configured ROS reagent was added to the cells and incubated at 37°C for 30 min. The cells were then digested with trypsin and collected, before being washed twice with PBS. A flow cytometer (FACSCalibur, BD Biosciences, San Jose, CA, USA) was used to determine the average fluorescence intensity of the sample.

### 2.9. Mitochondrial Membrane Potential Detection

After intervention, according to the manufacturer's instructions, the prepared JC-1 (Beyotime) reaction solution was added to the cell sample and incubated at 37°C for 30 minutes. The cells were washed twice in the configured JC-1 buffer. The cells were then mounted and photographed under a fully automatic fluorescence microscope (BX63, Olympus, Tokyo, Japan). The fluorescence intensity was analyzed using ImageJ software (version 1.51j8; NIH, Bethesda, MA, USA).

### 2.10. Iron Detection

We used an iron detection kit (ab83366, Abcam, Cambridge, UK) to detect ferrous and ferric ions in the samples. After intervention, the cell sample was mixed with the corresponding detection reagent, according to the manufacturer's instructions, and incubated at 37°C for 30 min. The iron probe was added, mixed, and incubated for 60 min at 37°C in the dark. Immediately thereafter, the absorbance of each well was detected at a wavelength of 593 nm using a microplate reader.

### 2.11. Immunofluorescence Detection

After intervention, the cell sample was washed once with PBS and fixed with universal tissue fixative (Servicebio, Wuhan, China) for 15 minutes, and 0.3% Triton-X-100 (Servicebio) was used to break the membrane for 8 minutes. The sample was blocked using 5% bovine serum albumin (BSA) (Servicebio) for 30 minutes, before adding anti-BECN1 (11306-1-AP, 1 : 100, Proteintech, Rosemont, IL, USA) and anti-GPX4 (14432-1-AP, 1 : 100, Proteintech) primary antibodies and incubating at 4°C for 12 hours. Next, fluorescent secondary antibodies (4412, Cell Signaling Technology (CST), Danvers, MA, USA) were added and incubated for 30 min at room temperature. After washing, the cells were mounted on slides and photographed under a fully automatic fluorescence microscope. The fluorescence intensity was analyzed using ImageJ software.

### 2.12. Quantitative Real-Time Reverse Transcription PCR (qRT-PCR)

After intervention, the cell sample was incubated with Trizol (15596026, Thermo Fisher) to extract the total RNA. Then, a PrimeScript™ RT Master Mix (Perfect Real Time; RR036A, Takara, Shiga, Japan) was used to reverse transcribe the total RNA into cDNA. Finally, a TB Green® Premix Ex Taq™ II (TliRNaseH Plus) (RR820A, Takara) kit was used to detect the relative expression of the target genes. The relative gene expression level of the target genes was determined using the formula: ratio = (1 + *E*_target_)^ΔCt target(control − expt)/^(1 + *E*_reference_)^ΔCt reference(control − expt)^. The specific steps were described in detail in our previous study [3]. The primer sequences of the target genes were as follows: *GAPDH* forward 5′-GTCTCCTCTGACTTCAACAGCG-3′ and reverse 5′-ACCACCCTGTTGCTGTAGCCAA-3′; *BECN1* forward 5′-CTGGACACTCAGCTCAACGTCA-3′ and reverse 5′-CTCTAGTGCCAGCTCCTTTAGC-3′; *GPX4* forward 5′-ACAAGAACGGCTGCGTGGTGAA-3′ and reverse 5′-GCCACACACTTGTGGAGCTAGA-3′; and *NCOA4* forward 5′-GCTTGCTATTGGTGGAGTTCTCC-3′ and reverse 5′-GCCATACCTCACGGCTTCTAAG-3′.

### 2.13. Western Blotting

After intervention, the total proteins of the cell sample were extracted using radioimmunoprecipitation assay (RIPA) lysis buffer (Beyotime). The protein sample was then subjected to protein gel electrophoresis. After transfer and blocking, the proteins on the membrane were incubated with primary antibodies recognizing GPX4 (14432-1-AP, 1 : 1000, Proteintech), acyl-CoA synthetase long-chain family member 4 (ACSL4) (22401-1-AP, 1 : 1000, Proteintech), solute carrier family 7 member 11 (xCT) (26864-1-AP, 1 : 1000, Proteintech), transferrin receptor (TFR1/CD71) (10084-2-AP, 1 : 1000, Proteintech), Ferritin Light Chain (FTL) (10727-1-AP, 1 : 1000, Proteintech), BECN1 (11306-1-AP, 1 : 2000, Proteintech), microtubule-associated protein 1 light chain 3 alpha (LC3) (14600-1-AP, 1 : 2000, Proteintech), NCOA4 (DF4255, 1 : 1000, Affbiotech, Jiangsu, China), sequestosome 1 (P62) (18420-1-AP, 1 : 2000, Proteintech), and glyceraldehyde-3-phosphate dehydrogenase (GAPDH) (60004-1-Ig, 1 : 50000, Proteintech) at 4°C for 12 hours. The membranes were washed four times with Tris-buffered saline (TBS). The membranes were then incubated with the secondary antibody (SA00001-1/SA00001-2, 1 : 20000, Proteintech) at room temperature for 1 hour and then washed four times. The immunoreactive proteins on the membrane were visualized using a chemiluminescence imager (ChemiDoc™ Touch, Bio-Rad, Hercules, CA, USA). Finally, ImageJ software was used to analyze the gray value of the protein bands.

### 2.14. Transmission Electron Microscopy

After intervention, the medium was removed from the cells and the electron microscope fixative (Servicebio) was added and incubated for 15 minutes. The cells were scraped into a centrifuge tube, fixed using osmic acid at 4°C for 12 hours, and then dehydrated. After a series of steps, such as infiltration, embedding, sectioning, and uranium-lead double staining, we observed the cells using a transmission electron microscope (Tecnai G2 20 TWIN, FEI, Karlsruhe, Germany).

### 2.15. Silver Nitrate Staining

The wax blocks from each group of rats were sliced, dewaxed, washed with distilled water, and immersed in silver nitrate dye solution for 10 minutes under ultraviolet light irradiation. The samples were then washed with distilled water for 3 minutes and passed through sodium thiosulfate and hematoxylin dye solution. After a series of treatments, such as hydrochloric acid alcohol differentiation and eosin staining, the slides were sealed with neutral gum and observed under a fully automatic fluorescence microscope.

### 2.16. Immunohistochemistry

Sections were dewaxed, soaked in gradient alcohol for 5 minutes, and placed in citric acid repair solution at high temperature and high pressure for 2 hours. The sections were then washed three times with TBS and incubated with 3% H_2_O_2_ at room temperature for 20 minutes. After washing with TBS, 10% goat serum was added and incubated for 20 minutes. Next, the corresponding primary antibody recognizing NCOA4 (DF4255, 1 : 100, Affbiotech) was added and incubated for 12 hours at 4°C. After washing three times with TBS, the sections were incubated for 25 minutes with horse radish peroxidase- (HRP-) goat anti-rat lgG (H+L) conjugate (ANT058, AntGene, Wuhan, China). Diaminobenzidine was then added to develop the color, after which the sections on slides were stained with hematoxylin, sealed with neutral resin, and observed under a fully automatic fluorescence microscope. NCOA4-positive staining was analyzed using ImageJ software.

### 2.17. Statistical Analysis

All data are expressed as mean ± standard error of the mean (SEM) of at least three independent experiments. All statistical analyses were performed using GraphPad Prism software version 7.0 (GraphPad Software Inc., La Jolla, CA, USA). Comparisons between two groups were determined using Student's *t*-test, while comparisons between multiple groups were analyzed using one-way analysis of variance (ANOVA), followed by Tukey's post hoc tests. *P* values < 0.05 were considered statistically significant.

## 3. Results

### 3.1. Oxalate Induces Excessive Autophagy in HK-2 Cells

**Figure 1:**
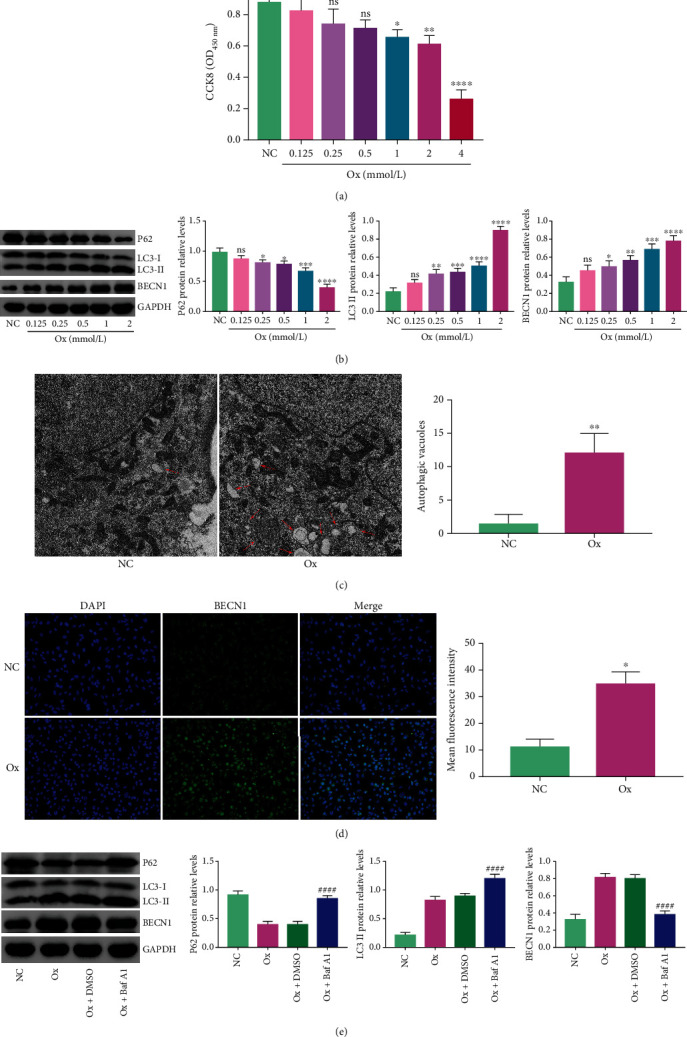
Oxalate induced excessive autophagy in HK-2 cells. The concentration of oxalate in the Ox group was 2 mmol/L, the concentration of Baf A1 was 50 nmol/L, and the intervention time was 24 hours. The CCK8 results of cell viability under different concentrations of oxalate (a). The bands and histograms of western blotting represent the relative levels of BECN1, p62, and LC3II under different concentrations of oxalate (b). We observed the number of autophagic vesicles in the two groups under a 10 K projection electron microscope (c). The automatic microscope had a 200-fold view to observe the fluorescence intensity of the two groups. The stronger the fluorescence intensity, the higher the level of BECN1 (d). The bands and histograms of western blotting represent the relative levels of BECN1, LC3II, and p62 proteins of the four groups compared with that of GAPDH (e). Data are presented as the means ± SEM from three independent experiments. ^∗^*P* < 0.05, ^∗∗^*P* < 0.01, ^∗∗∗^*P* < 0.001, and ^∗∗∗∗^*P* < 0.0001 versus the negative control (NC) group. ^#^*P* < 0.05, ^##^*P* < 0.01, ^###^*P* < 0.001, and ^####^*P* < 0.0001 versus the Ox+DMSO group. DMSO: dimethyl sulfoxide.

In this study, to verify the occurrence of autophagy, we detected autophagic vesicles and autophagy-related proteins in the NC group and the Ox group. Firstly, the CCK8 experiment showed that 4 mmol/L oxalate had an extreme killing effect on HK-2 cells (Figure 1(a)). Moreover, we detected the expression of autophagy marker proteins in HK-2 cells under different concentrations of oxalate. Compared with the NC group, the protein levels of BECN1 and LC3II in the Ox group were significantly increased, while the protein level of p62 was significantly decreased. The differential abundance of this protein increased with the increase in oxalate concentration, especially in 2 mmol/L oxalate (Figure 1(b)). Therefore, we chose 2 mmol/L oxalate as the concentration for subsequent experiments. Then, compared with that in the NC group, the number of intracellular autophagic vesicles in the Ox group increased significantly (Figure 1(c)), and the protein level of BECN1 in the cells increased significantly (Figure 1(d)). In addition, we used bafilomycin A1 (Baf A1) to verify the changes to autophagy proteins. The results showed that compared with those in the Ox+DMSO group, the protein levels of LC3II and p62 in the Ox+Baf A1 group were significantly increased, and BECN1 was significantly decreased (Figure 1(e)). The above results indicated that oxalate could induce excessive autophagy in HK-2 cells.

### 3.2. Oxalate Induces Oxidative Stress Damage in HK-2 Cells

**Figure 2:**
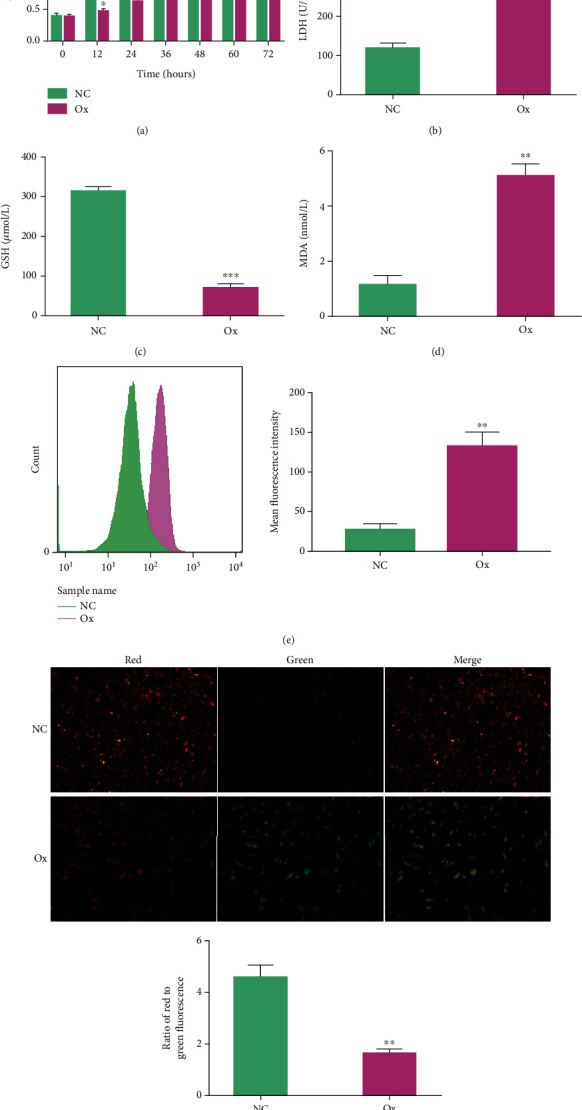
Oxalate induced oxidative stress damage in HK-2 cells. The concentration of oxalate was 2 mmol/L, and the intervention time was 24 hours. The cell viability of the NC group and the Ox group was observed at different times (a), and the LDH content (b), GSH content (c), MDA content (d), and ROS levels were compared after 24 hours of intervention in the NC group and Ox group (e). The mitochondrial membrane potential (f): fluorescence images were taken under a 200-fold field of view of a fully automatic microscope, and the ratio of red to green fluorescence represents the magnitude of the mitochondrial membrane potential. Data are presented as the means ± SEM from three independent experiments. ^∗^*P* < 0.05, ^∗∗^*P* < 0.01, ^∗∗∗^*P* < 0.001, and ^∗∗∗∗^*P* < 0.0001 versus the NC group.

To verify that oxalate can induce oxidative stress damage in HK-2 cells, we used 2 mmol/L oxalate to treat HK-2 cells for 24 hours and then tested cell viability and LDH in the NC group and the Ox group; we also performed GSH tests and MDA tests and assessed ROS levels and the mitochondrial membrane potential. The results showed that, compared with the NC group, the cell viability of the Ox group decreased significantly in an intervention time-dependent manner (Figure 2(a)). The LDH, MDA, and ROS levels increased significantly (Figures 2(b), 2(d), and 2(e)). The GSH content and mitochondrial membrane potential level decreased significantly (Figures 2(c) and 2(f)). These results confirmed that after 24 hours of treatment of HK-2 cells with 2 mmol/L oxalate, the cells experienced oxidative stress damage, such as decreased cell viability, the accumulation of lipid peroxides, increased ROS levels, and decreased mitochondrial membrane potential.

### 3.3. Oxalate Induced Ferroptosis in HK-2 Cells

**Figure 3:**
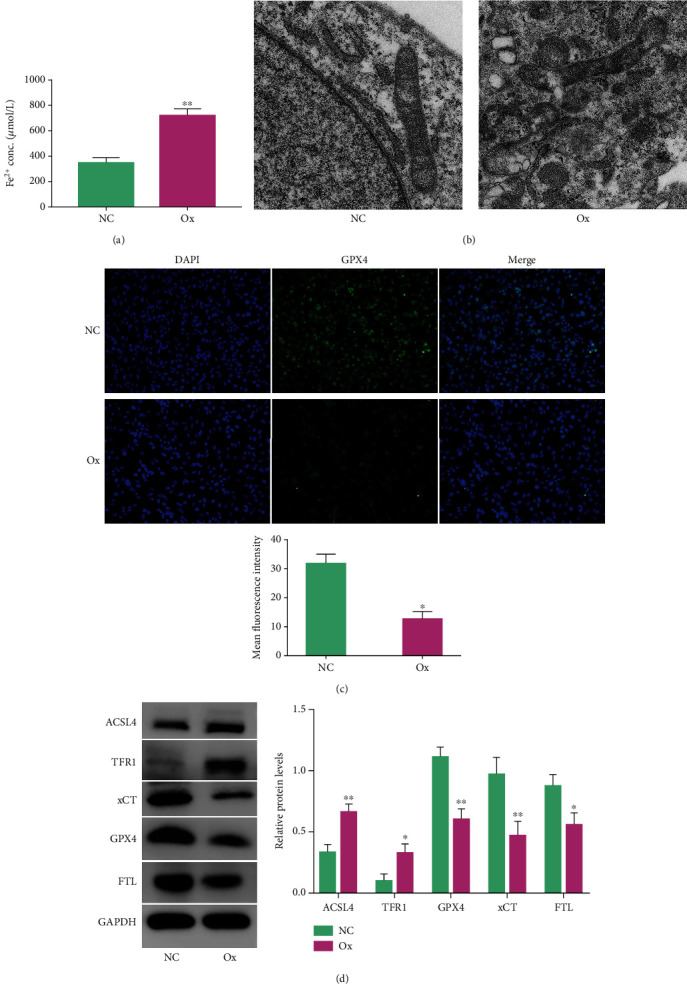
Oxalate induced ferroptosis in HK-2 cells. The concentration of oxalate was 2 mmol/L, and the intervention time was 24 hours. We compared the Fe^2+^ content of the NC group and the Ox group after 24 hours of intervention (a). The mitochondrial damage of the two groups under a 10 K field of a projection electron microscope (b). The fluorescence intensity of the two groups under a 200× field of the automatic microscope; the stronger the fluorescence intensity, the higher the level of GPX4 (c). The bands and histograms of western blotting represent the relative levels of ACSL4, TFR1, FTL, GPX4, and xCT proteins in the two groups relative to GAPDH (d). Data are presented as the means ± SEM from three independent experiments. ^∗^*P* < 0.05, ^∗∗^*P* < 0.01, ^∗∗∗^*P* < 0.001, and ^∗∗∗∗^*P* < 0.0001 versus the NC group.

Ferroptosis is an iron-dependent cell death method characterized by the loss of GPX4 activity and the accumulation of lipid peroxides and ROS [16]. Therefore, to verify that oxalate can induce ferroptosis in HK-2 cells, we treated HK-2 cells with 2 mmol/L oxalate for 24 hours and then tested the Fe^2+^ content in the NC group and the Ox group, observed the mitochondria under a transmission electron microscope, and performed immunofluorescence and western blotting detection. The results showed that compared with the NC group, the Fe^2+^ content of the Ox group increased significantly (Figure 3(a)). The mitochondria in the cells showed outer membrane rupture, the mitochondria were swollen, and the mitochondrial cristae had disappeared or were twisted (Figure 3(b)). The level of GPX4 in the cells decreased significantly (Figure 3(c)). The protein levels of ACSL4 and TFR1 in the cell increased significantly, and the protein levels of xCT, GPX4, and FTL decreased significantly (Figure 3(d)). The above results confirmed that oxalate could induce ferroptosis in HK-2 cells.

### 3.4. Knockdown of *BECN1* Expression in HK-2 Cells Can Reduce Ferroptosis of Oxalate-Induced Cells

**Figure 4:**
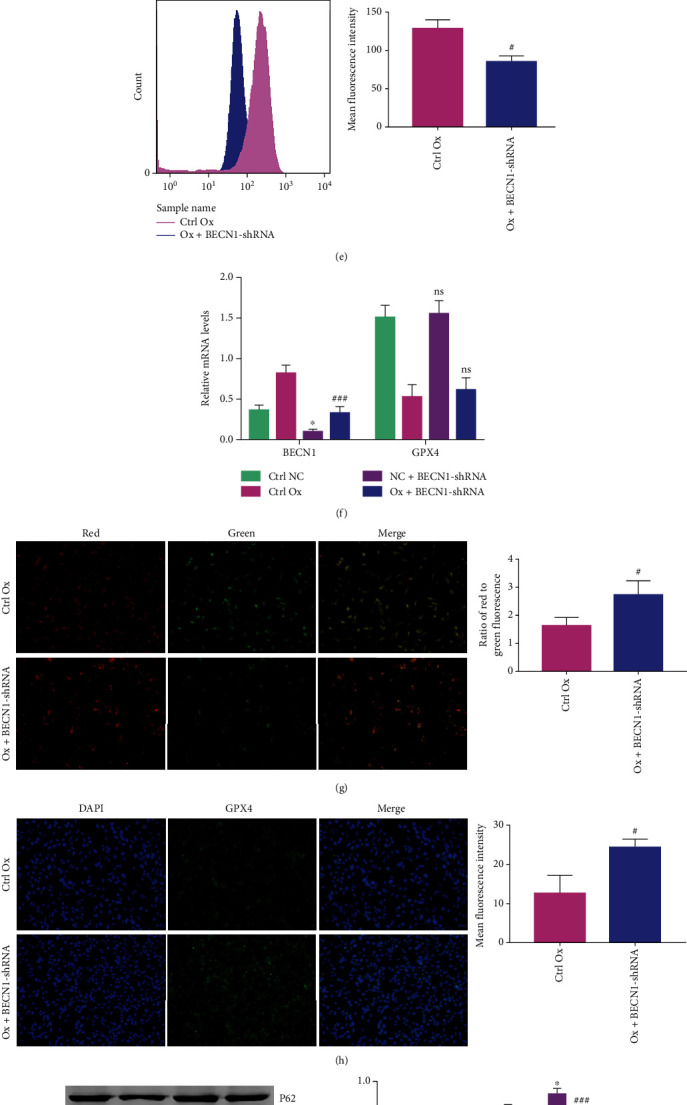
Knockdown of the expression of *BECN1* in HK-2 cells can reduce the ferroptosis of oxalate-induced cells. The concentration of oxalate was 2 mmol/L, and the intervention time was 24 hours. Among the four groups of cells, we compared the levels of BECN1 protein relative to GADPH levels (a), the MDA content (b), the GSH content (c), the Fe^2+^ content (d), the ROS levels (e), the mitochondrial membrane potential (g), the GPX4 protein levels (h), the BECN1 and GPX4 mRNA levels (relative to GADPH expression) (f), and the levels of P62, LC3 II, ACSL4, TFR1, TFH1, GPX4, and xCT proteins relative to GADPH levels (i). The fluorescence images were all taken under a 200× field of view under an automatic microscope. Data are presented as the means ± SEM from three independent experiments. ^#^*P* < 0.05, ^##^*P* < 0.01, ^###^*P* < 0.001, and ^####^*P* < 0.0001 versus the ctrl Ox group. ^∗^*P* < 0.05, ^∗∗^*P* < 0.01, ^∗∗∗^*P* < 0.001, and ^∗∗∗∗^*P* < 0.0001 versus the ctrl NC group. ns: not significant.

To study the role of autophagy in oxalate-induced ferroptosis, we used *BECN1*-shRNA plasmids to knockdown *BECN1* expression in HK-2 cells. Subsequently, we detected MDA, GSH, Fe^2+^, ROS, and the mitochondrial membrane potential, performed immunofluorescence and western blotting to detect the expression of ferroptosis-related proteins, and performed qRT-PCR to detect intracellular *GPX4* and *BECN1* mRNA expression. Successful knockdown of *BECN1* was confirmed using western blotting (Figure 4(a)) and qRT-PCR (Figure 4(f)). Compared with those in the control Ox group, the levels of MDA (Figure 4(b)) and Fe^2+^ (Figure 4(d)) and ROS (Figure 4(e)) in the Ox+BECN1-shRNA group decreased, the GSH content (Figure 4(c)) and mitochondrial membrane potential (Figure 4(g)) increased, the intracellular ACSL4 and TFR1 protein levels decreased, and FTL, GPX4 (Figures 4(h) and 4(i)), and xCT protein levels increased significantly (Figure 4(i)). Interestingly, the results of qRT-PCR showed that compared with that in the control Ox group, the difference in *GPX4* mRNA expression in the Ox+BECN1-shRNA group was not statistically significant (Figure 4(f)); therefore, we hypothesized that during oxalate-induced ferroptosis of HK-2 cells, BECN1 directly consumes GPX4 protein or blocks the translation of *GPX4* in some way, rather than affecting mRNA transcription. The above results showed that knocking down the expression of *BECN1* in HK-2 cells reduced the oxalate-induced ferroptosis of cells, and this effect was not produced by affecting the transcription of *GPX4* mRNA.

### 3.5. Overexpression of *BECN1* in HK-2 Cells Can Exacerbate Ferroptosis in Cells Treated with Oxalate

**Figure 5:**
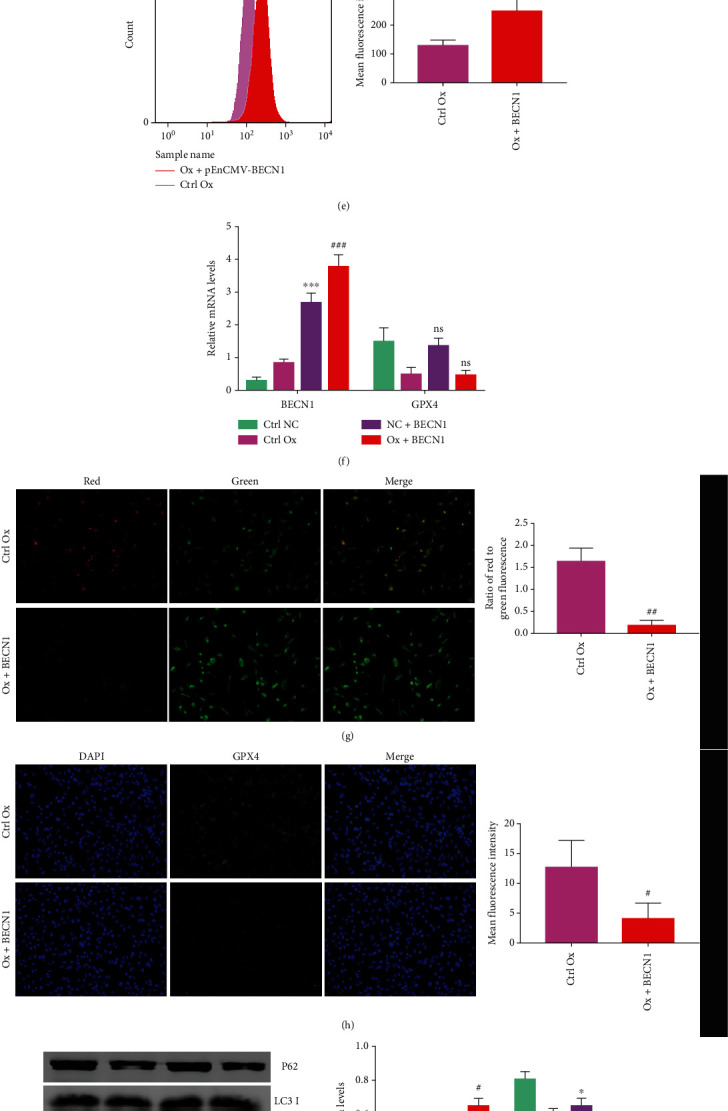
Overexpression of *BECN1* in HK-2 cells can exacerbate the ferroptosis of cells induced by oxalate. The concentration of oxalate was 2 mmol/L, and the intervention time was 24 hours. Among the four groups of cells, we compared the levels of BECN1 protein relative to GADPH levels (a), the MDA content (b), the GSH content (c), the Fe^2+^ content (d), the ROS levels (e), the mitochondrial membrane potential (g), GPX4 protein level of the ctrl Ox and Ox+BECN1 groups (h), and the expression of *BECN1* and *GPX4* mRNA in the four groups of cells relative to that of GAPDH (f). We compared the levels of P62, LC3 II, ACSL4, TFR1, TFH1, GPX4, and xCT proteins in the four groups of cells relative to the GAPDH levels (i). The fluorescence images were all taken under a 200× field of view under an automatic microscope. Data are presented as the means ± SEM from three independent experiments. ^#^*P* < 0.05, ^##^*P* < 0.01, ^###^*P* < 0.001, and ^####^*P* < 0.0001 versus the ctrl Ox group; ^∗^*P* < 0.05, ^∗∗^*P* < 0.01, ^∗∗∗^*P* < 0.001, and ^∗∗∗∗^*P* < 0.0001 versus the ctrl NC group. ns: not significant.

To further study whether the overexpression of *BECN1* in HK-2 cells affected the ferroptosis of cells induced by oxalate, we used the pEnCMV-BECN1 plasmid to overexpress *BECN1* in HK-2 cells and detected MDA, GSH, Fe^2+^, ROS, and the mitochondrial membrane potential, as well as performing immunofluorescence and western blotting to detect the expression of ferroptosis-related proteins. qRT-PCR was used to detect the mRNA expression of *GPX4* and *BECN1* in cells. Western blotting (Figure 5(a)) and qRT-PCR (Figure 5(f)) verified the successful overexpression of *BECN1*. Compared with those in the control Ox group, the levels of MDA (Figure 5(b)), Fe^2+^ (Figure 5(d)), and ROS (Figure 5(e)) increased significantly in the Ox+BECN1 group. The GSH (Figure 5(c)) content and mitochondrial membrane potential (Figure 5(g)) decreased, intracellular ACSL4 and TFR1 protein levels increased, and FTL, GPX4 (Figures 5(h) and 5(i)), and xCT protein levels decreased significantly in the Ox+BECN1 group (Figure 5(i)). Consistent with the results in the Ox+BECN1-shRNA group, overexpression of *BECN1* did not affect the transcription of *GPX4* mRNA (Figure 5(f)). The above results showed that overexpression of *BECN1* in HK-2 cells exacerbated the ferroptosis of oxalate-induced cells, and this effect was not caused by altering the transcription of *GPX4* mRNA.

### 3.6. NCOA4 Is Highly Expressed in Cell and Animal Models of Stone Formation

**Figure 6:**
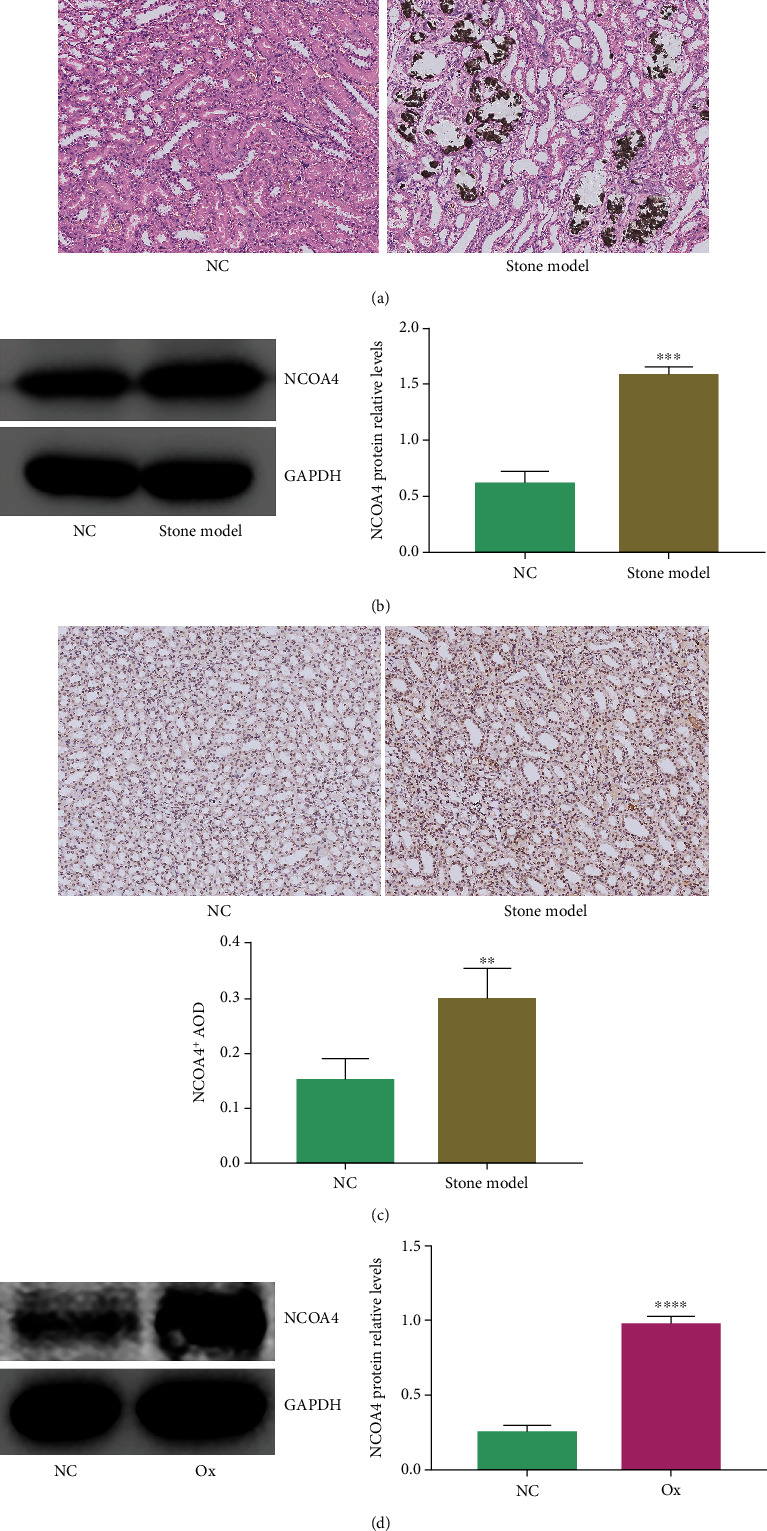
NCOA4 is highly expressed in cell and animal models of stone formation. The concentration of oxalate was 2 mmol/L, and the intervention time was 24 hours. We compared the degree of calcium deposition in the renal tubules of the control group and the stone model group (a); the dark brown deposits shown in the image represent calcium deposits. We compared the level of NCOA4 protein relative to that of GAPDH in the two groups of kidneys (b). Images of immunohistochemical staining were used to compare the NCOA4-positive staining in the renal tubules of the two groups (c); the higher the proportion of positive staining, the higher the protein level. Images in (a) and (c) were all taken under a 200× field of view under an automatic microscope. The levels of NCOA4 proteins relative to the GAPDH in the NC and Ox group (d). Data are presented as the means ± SEM from three independent experiments. ^∗^*P* < 0.05, ^∗∗^*P* < 0.01, ^∗∗∗^*P* < 0.001, and ^∗∗∗∗^*P* < 0.0001 versus the NC group.

NCOA4 is a selective cargo receptor, and NCOA4-mediated ferritin phagocytosis can maintain intracellular iron homeostasis by depleting intracellular ferritin [23]. To verify whether the ferroptosis caused by excessive autophagy in the kidney stone model is related to the abnormal expression of NCOA4, we detected the expression of NCOA4 by immunohistochemistry and western blotting in cell and animal models of stone formation. The results showed that compared with that in the NC group, the calcium deposits in the renal tubules of the stone model group increased significantly (Figure 6(a)), which indicated the successfully construction of the rat kidney stone model. The protein expression of NCOA4 in the renal tubules of the stone model group increased significantly (Figures 6(b) and 6(c)). Compared with that in the NC group, the protein expression of NCOA4 in the Ox group increased significantly (Figure 6(d)). These results indicated that NCOA4 is highly expressed in cell and animal models of stone formation.

### 3.7. NCOA4 Might Be a Key Factor in the Process of Ferroptosis Induced by Oxalate-Activated Autophagy in HK-2 Cells

**Figure 7:**
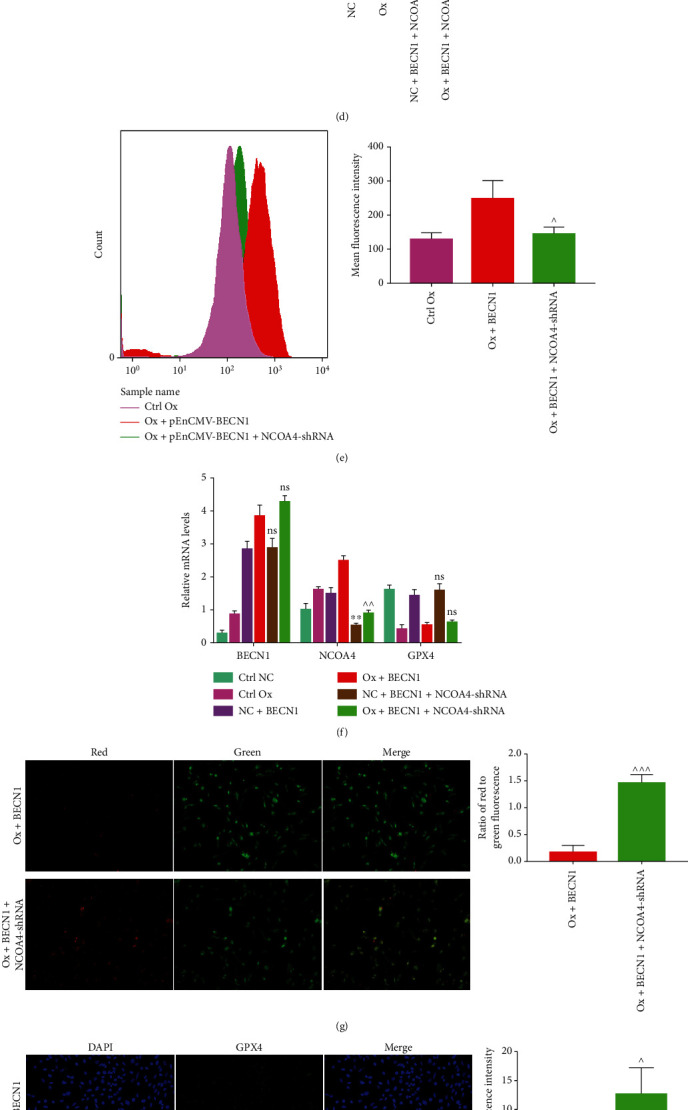
NCOA4 might be a key factor for oxalate induction of excessive autophagy in HK-2 cells to cause ferroptosis of cells. The concentration of oxalate was 2 mmol/L, and the intervention time was 24 hours. Among the six groups of cells, we compared the levels of BECN1 and NCOA4 proteins relative to GADPH levels (a), the MDA content (b), the GSH content (c), the Fe^2+^ content (d), the ROS levels (e) of the two groups Ox+BECN1 and Ox+BECN1+NCOA4-shRNA, the mitochondrial membrane potential level (g), the protein levels of GPX4 (h), the expression of *BECN1*, *GPX4*, and *NCOA4* mRNA in the six groups of cells relative to that of *GAPDH* (f), and the protein levels of P62, LC3 II, ACSL4, TFR1, TFH1, GPX4, and xTC in the six groups of cells (i). The fluorescence images were all taken under a 200× field of view under an automatic microscope. Data are presented as the means ± SEM from three independent experiments. ^^*P* < 0.05, ^^*P* < 0.01, ^^^*P* < 0.001, and ^^^^^^*P* < 0.0001 versus the Ox+BECN1 group; ^∗^*P* < 0.05, ^∗∗^*P* < 0.01, ^∗∗∗^*P* < 0.001, and ^∗∗∗∗^*P* < 0.0001 versus the NC+BECN1 group; ^#^*P* < 0.05, ^##^*P* < 0.01, ^###^*P* < 0.001, and ^####^*P* < 0.0001 versus the ctrl NC group. ns: not significant.

To verify whether high expression of NCOA4 plays a role in oxalate-induced excess autophagy in HK-2 cells leading to ferroptosis, we used the pEnCMV-BECN1 plasmid and the NCOA4-shRNA plasmid to overexpress *BECN1* and knockdown *NCOA4* in HK-2 cells, followed by the detection of MDA, GSH, Fe^2+^, ROS, and the mitochondrial membrane potential. Immunofluorescence and western blotting were used to detect the expression of ferroptosis-related proteins, and qRT-PCR was used to detect the mRNA expression of *GPX4*, *BECN1*, and *NCOA4* in cells. Western blotting (Figure 7(a)) and qRT-PCR (Figure 7(f)) verified the successful knockdown of *NCOA4*. Compared with those in the Ox+BECN1 group, the cellular levels of MDA (Figure 7(b)), Fe^2+^ (Figure 7(d)), and ROS (Figure 7(e)) decreased in the Ox+BECN1+NCOA4-shRNA group. The GSH content (Figure 7(c)) and mitochondrial membrane potential (Figure 7(g)) increased, the protein levels of ACSL4 and TFR1 decreased, and the protein levels of FTL, GPX4 (Figures 7(h) and 7(i)), and xCT increased significantly in the Ox+BECN1+NCOA4-shRNA group (Figure 7(i)). In addition, the difference in *GPX4* mRNA expression between the two groups of cells was not statistically significant (Figure 7(f)). The above results showed that knockdown of *NCOA4* could largely reverse the aggravating effect of *BECN1* overexpression on cell ferroptosis, which suggested that NCOA4 might be a key factor in the process of ferroptosis induced by oxalate-activated autophagy in HK-2 cells.

## 4. Discussion

Calcium oxalate monohydrate stones are the most common type of kidney stones [24]. In addition, oxalate is the most important factor in the accumulation of urinary liquid crystals and is also an important cause of proximal and distal kidney tubular cell damage [8–10]. Therefore, studying the toxic effect of oxalate on renal tubular epithelial cells is vitally important to determine the mechanism of kidney stone formation. The present study provided evidence that oxalate activates autophagy to induce ferroptosis in HK-2 cells, in which NCOA4 is hypothesized to act as a bridge.

Autophagy is a form of cell self-degradation [25]. When misfolded proteins, damaged organelles, and foreign pathogenic microorganisms appear in the cell, the cell produces autophagic vesicles to swallow these harmful substances, which then fuse with lysosomes. Lysosomal acid proteases then degrade the autophagosome contents, and the degraded products are exported back to the cytoplasm for reuse, which ultimately supports healthy cell growth [26, 27]. However, excessive autophagy can cause diseases, such as epilepsy [28], femoral head necrosis [29], myocardial injury [30], and pancreatic cancer [31]. *BECN1* is a key factor that controls autophagy activity. Increased expression of *BECN1* is regarded as a marker for the activation of autophagy [32]. In addition, the formation of autophagosomes is accompanied by the conversion of LC3 I to LC3 II. Therefore, LC3 expression has also become a marker of autophagy, in which the ratio of LC3 II/LC3 I also represents the level of autophagy [33]. Western blotting and immunofluorescence experiments showed that after 24 hours of oxalate intervention in HK-2 cells, the amount of BECN1 and the ratio LC3 II/LC3 I in the cells increased. In addition, we used Baf A1 to verify the changes in autophagy protein levels. The increase in LC3 II and p62 levels in the Ox+Baf A1 group confirmed the increase in autophagy flux in HK-2 cells treated with oxalate. We also observed a significant increase in the number autophagic vesicles in the oxalate-treated cells using transmission electron microscopy. Our results confirmed that oxalate can induce excessive autophagy in renal tubular epithelial cells and reduce cell viability.

Since Dixon et al. first proposed the concept of ferroptosis in 2012 [16], researchers have discovered that ferroptosis is present in many diseases, such as Alzheimer's disease [34], breast cancer [35], pancreatic cancer [36], ovarian cancer [37], and acute kidney injury [38]. Lipid peroxides and iron accumulation, as well as mitochondrial failure, are prominent features of ferroptosis [16]. In the present study, we used transmission electron microscopy to observe the changes in HK-2 cell mitochondria after oxalate treatment. The mitochondria became swollen, the cristae shrank, and the mitochondrial membrane was broken. In addition, the levels of ROS and Fe^2+^ accumulated significantly in the cells of the Ox group. Impaired GSH metabolism is also an important cause of ferroptosis in cells. GSH can reduce toxic lipid peroxides to nontoxic fatty alcohols under the catalysis of GPX4. When GSH metabolism is impaired, excessive accumulation of toxic lipids can cause cellular ferroptosis [39]. Therefore, we tested the GSH and MDA levels in the cells and found that compared with that in the NC group, the GSH level in the Ox group decreased significantly and the MDA level increased significantly. In addition, *Gpx4* knockdown in mice induced kidney iron-mediated death and acute renal failure, confirming that GPX4 is a key regulator of ferroptosis [40]. In our study, we used immunofluorescence and western blotting to detect the protein levels of GPX4 in each group and found that compared with the NC group, the levels of GPX4 in the Ox group decreased significantly. In addition, we detected other ferroptosis-related proteins, such as ACSL4, TFR1, FTL, and xCT. The results showed that ACSL4 and TFR1 levels increased and FTL and xCT levels decreased. The above results confirmed that oxalate could induce ferroptosis in HK-2 cells.

The induction of autophagy can induce ferroptosis, and some researchers believe that ferroptosis is a type of autophagy-dependent cell death [18]. Knockout of *Atg5* (encoding autophagy-related 5) and *Atg7* (encoding autophagy-related 7) in mouse embryonic fibroblasts induced ferroptosis [41]. In this study, we explored the role of autophagy activation in oxalate-induced ferroptosis in HK-2 cells by knockdown and overexpression of the key autophagy gene *BECN1*. The results showed that knockdown of *BECN1* in HK-2 cells significantly reduced oxalate-induced ferroptosis, reduced the lipid peroxide level in the cells significantly, decreased ROS levels significantly, and increased the mitochondrial membrane potential significantly. *BECN1* knockdown also increased the GPX4 protein level significantly. By contrast, *BECN1* overexpression in HK-2 cells aggravated the oxalate-induced toxicity of ferroptosis in HK-2 cells. Notably, neither knockdown nor overexpression of *BECN1* affected *GPX4* mRNA expression in HK-2 cells significantly. Therefore, the above results indicated that oxalate can induce ferroptosis in HK-2 cells by activating autophagy, and this effect is not caused by altering the transcription of *GPX4*.

NCOA4 is a selective cargo receptor, and the phagocytosis of ferritin mediated by NCOA4 can maintain iron homeostasis in the cell by depleting ferritin [23]. Under normal circumstances, iron in cells mainly exists in the form of nontoxic ferritin, which is composed of ferritin heavy and light chains. When the human body needs iron, autophagy is induced, in which NCOA4 combines with ferritin and transports it to the lysosome for digestion, which finally releases free iron [42]. Studies have shown that knockdown of the expression of *NCOA4* or *ATG5* can inhibit the degradation of ferritin induced by erastin and its subsequent ferroptosis [41]. In this study, we performed *BECN1* gene overexpression and *NCOA4* gene knockdown in HK-2 cells. Compared with the *BECN1* overexpression group, the cotransfection group showed lower levels of ROS, Fe^2+^, and MDA, decreased levels of the ferroptosis-related proteins ACSL4 and TFR1, and increased levels of GPX4, FTL, and xCT. This suggested that knockdown of *NCOA4* can reverse the effects of overexpression of *BECN1* and aggravate the toxicity of ferroptosis in HK-2 cells induced by oxalate.

## 5. Conclusions

In summary, we confirmed that oxalate can activate autophagy and lead to ferroptosis in HK-2 cells. Our results prompted us to hypothesize that NCOA4 might act as a bridge in this process. However, because of insufficient experimental conditions and other reasons, we did not conduct animal experiments to explore this process. Instead, we used genetic engineering techniques such as knockdown and overexpression of *BECN1* and knockdown of *NCOA4*, to activate autophagy in HK-2 cells induced by oxalate. The mechanism of ferroptosis in cells was explored in more detail, revealing the role of autophagy-ferroptosis in the formation of kidney stones and providing new ideas for the prevention and treatment of kidney stones in the future.

## Data Availability

The original data is available upon reasonable request.
